# Homozygous variants in the *HEXB* and *MBOAT7* genes underlie neurological diseases in consanguineous families

**DOI:** 10.1186/s12881-019-0907-7

**Published:** 2019-12-18

**Authors:** Shazia Khan, Lettie E. Rawlins, Gaurav V. Harlalka, Muhammad Umair, Asmat Ullah, Shaheen Shahzad, Muhammad Javed, Emma L. Baple, Andrew H. Crosby, Wasim Ahmad, Asma Gul

**Affiliations:** 1 Department of Biological Sciences, International Islamic University Islamabad, H-10, Islamabad, 44000 Pakistan; 2 Medical Research, RILD Wellcome Wolfson Centre (Level 4), Royal Devon and Exeter NHS Foundation Trust, Exeter, Devon EX2 5DW UK; 3 Department of Biochemistry, Faculty of Biological Sciences, Quaid-i-Azam University, Islamabad, Pakistan; 4 Peninsula Clinical Genetics Service, Royal Devon & Exeter Hospital (Heavitree), Gladstone Road, Exeter, EX1 2ED UK; 5 Rajarshi Shahu College of Pharmacy, Malvihir Buldana, Maharashtra, Buldana, 443001 India; 6 Medical Genomics Research Department, King Abdullah International Medical Research Center (KAIMRC), King Saud bin Abdulaziz University for Health Sciences, Ministry of National Guard–Health Affairs (MNGHA), P.O. Box 3660, Riyadh, 11481 Kingdom of Saudi Arabia; 7 Department of Molecular Biology, Shaheed Zulfiqar Ali Bhutto Medical University, Islamabad, Pakistan; 8 National Institute for Genomics & Advanced Biotechnology, NARC, Islamabad, 45500 Pakistan

**Keywords:** Neurological disorder, *HEXB*, *MBOAT7*, Exome sequencing, Sandhoff disease, Pakistan

## Abstract

**Background:**

Neurological disorders are a common cause of morbidity and mortality within Pakistani populations. It is one of the most important challenges in healthcare, with significant life-long socio-economic burden.

**Methods:**

We investigated the cause of disease in three Pakistani families in individuals with unexplained autosomal recessive neurological conditions, using both genome-wide SNP mapping and whole exome sequencing (WES) of affected individuals.

**Results:**

We identified a homozygous splice site variant (NM_000521:c.445 + 1G > T) in the hexosaminidase B (*HEXB)* gene confirming a diagnosis of Sandhoff disease (SD; type II GM2-gangliosidosis), an autosomal recessive lysosomal storage disorder caused by deficiency of hexosaminidases in a single family. In two further unrelated families, we identified a homozygous frameshift variant (NM_024298.3:c.758_778del; p.Glu253_Ala259del) in membrane-bound O-acyltransferase family member 7 (*MBOAT7)* as the likely cause of disease. *MBOAT7* gene variants have recently been identified as a cause of intellectual disability (ID), seizures and autistic features.

**Conclusions:**

We identified two metabolic disorders of lipid biosynthesis within three Pakistani families presenting with undiagnosed neurodevelopmental conditions. These findings enabled an accurate neurological disease diagnosis to be provided for these families, facilitating disease management and genetic counselling within this population. This study consolidates variation within *MBOAT7* as a cause of neurodevelopmental disorder, broadens knowledge of the clinical outcomes associated with *MBOAT7*-related disorder, and confirms the likely presence of a regionally prevalent founder variant (c.758_778del; p.Glu253_Ala259del) in Pakistan.

## Background

Neurological disorders cause structural, functional, biochemical or electrical abnormalities in the nervous system, resulting in cognitive impairment, seizures, muscle weakness, paralysis, poor coordination and mood alteration. Neurological disorders are an increasing burden in developing countries due to improving life expectancy, urbanisation of the population and improved health care and diagnosis. A higher prevalence of intellectual disability (ID) and epilepsy have been identified within Pakistani populations compared with more economically developed countries [1, 2]. In Pakistan, 82.5% of the parents are blood relatives due to religious, economic, social and cultural reasons in different regions [3]. The *HEXB* gene encodes the hexosaminidase beta subunit, which forms a heterodimer with the alpha subunit in hexosaminidase A (HEXA) and a homodimer in hexosaminidase B (HEXB), which are important enzymes within neuronal membrane components responsible for GM2 ganglioside degradation. Sandhoff disease (SD)(MIM 268800) is an autosomal recessive lysosomal lipid storage disorder caused by biallelic variants within the *HEXB* gene, resulting in deficiency of HEXA and HEXB enzymes [4] and intralysosomal accumulation of GM2 ganglioside and related glycolipids within neurons. This leads to progressive destruction of the central nervous system (CNS); classical onset of SD occurs with onset of symptoms before 6 months of age of progressive psychomotor retardation, motor weakness, hyperreflexia, early blindness with cherry red spots, macrocephaly, and with death occurring by 3–5 years [5].

Recently homozygous pathogenic variants within *MBOAT7* have been identified in 16 families (15 consanguineous and 1 reported as non-consanguineous, although both parents were from the same village in Lebanon) as a cause of a neurodevelopmental disorder (autosomal recessive mental retardation type 57 (MIM 617188) characterized by seizures, moderate to severe ID with significant psychomotor retardation (several individuals are non-verbal and never walked, usually occurring with seizure onset), truncal hypotonia, appendicular hypertonia, features of autism spectrum disorder (ASD), below average head circumference and characteristic facial features [6–10]. The MBOAT protein family consists of five acyltransferases; lysophosphatidylinositol acyltransferase 1 (LPIAT1) encoded by the *MBOAT7* gene is known to transfer arachidonic acid (AA) from arachidonoyl-CoA to lysophosphatidylinositol [11]. Only one other MBOAT gene has been linked to human disease of brachydactyly-syndactyly syndrome in a single patient with a balanced translocation disrupting *MBOAT1* [12].

In the present study, we investigate three consanguineous Pakistani families with features of autosomal recessive neurological disorders in order to identify a precise molecular diagnosis using a combination of genome wide SNP mapping and whole exome sequencing (WES).

## Methods

### Ethics approval and consent to participate

This study was approved by the Institutional Ethical Review Board of International Islamic University, Islamabad, Pakistan. Written informed consent to participate was obtained from all individuals in the study or their parents.

Three families were recruited to the study from remote regions of Khyber Pakhtunkhwa (KP) province of Pakistan. Available affected and unaffected members of all families underwent clinical examination at local government hospitals with review of relevant medical records and blood samples were taken with informed consent.

### Genomic analysis

**Fig. 1:**
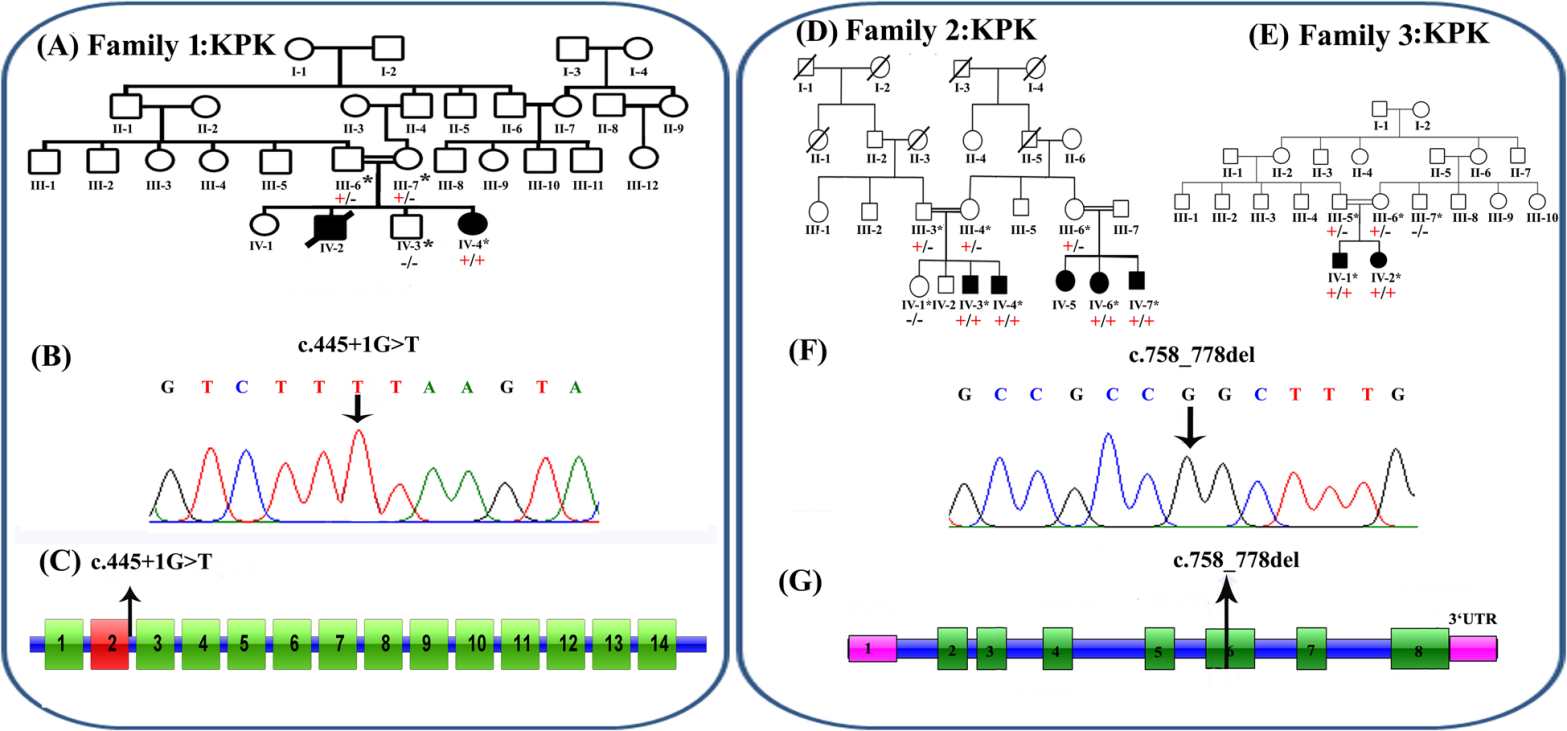
**a** Pedigree of family 1, with two affected siblings with Sandhoff disease (filled symbols). Genotype is shown in red under individuals (+, mutant; −, WT). * indicates samples available for analysis. The affected female was shown to be homozygous for the *HEXB* c.445 + 1G > T splice site variant. **b** Electropherogram showing the DNA sequence variant (*HEXB* c.445 + 1G > T) in a homozygous affected individual. **c** Schematic representation of *HEXB* exons and position of the genomic variant identified in this study. **d-e** Pedigrees of families 2 and 3, both from the Khyber Pakhtunkhwa province and with individuals affected with a neurodevelopmental disorder (filled symbols), within the same generation. Genotype is shown in red under individuals (+, mutant; −, WT). * indicates samples available for analysis. Six affected individuals were shown to be homozygous for the *MBOAT7* c.758_778del; p.(Glu253_Ala259del) variant (**f**) Electropherogram showing the DNA sequence variant (*MBOAT7* c.758_778del; p.(Glu253_Ala259del) in a homozygous affected individual (**g**) Schematic representation of *MBOAT7* exons and positions of the genomic variant identified in this study

Venous blood samples were collected in EDTA tubes (BD, Franklin Lakes, NJ, USA) from 15 individuals (shown with asterisks; Fig. 1) and DNA was extracted using the GenElute™ Blood Genomic DNA Kit (Merck) according to the manufacturer’s protocols and quantification using standard methods. WES was undertaken on DNA from a single affected individual of family A (IV-2) using the Agilent 2100 Bioanalyser/Illumina HiSeq2000 platform and exome enrichment was performed using SureSelect Human All ExonV4 (51 Mb) with a mean read depth of 30X. Reads were aligned (BWA-MEM), mate-pairs fixed and duplicates removed (Picard), inDel realignment and base quality recalibration performed (GATK). SNVs and InDels were detected using GATK HaplotypeCaller and custom annotation was performed using standard DNAnexus (DNAnexus Inc., Mountain View, CA; https://dnanexus.com). Data was filtered to identify rare non-synonymous exonic or splice variants, with a population frequency of < 0.01 in control databases (including the Genome Aggregation Database; gnomAD, the Exome Aggregation Consortium; ExAC, and the 1000 Genomes Project), and analysed considering the disease phenotype. Single-nucleotide polymorphism (SNP) genotyping was performed (HumanCytoSNP-12 v2.1 beadchip array, Illumina) in 3 affected individuals in family B. In silico prediction of variant pathogenicity was assessed using FATHMM (http://fathmm.biocompute.org.uk/), MutationTaster (http://www.mutationtaster.org/), Varsome (https://varsome.com/), DaNN (https://omictools.com/dann-tool), NNSplice (Berkeley, CA, USA), MutPred Splice (v1.3.2), MaxEnt, SKIPPY and Human Splice Finder (v2.4.1). Allele-specific primers were designed using Primer3 web software (http://frodo.wi.mit.edu/primer3/). PCR and dideoxy sequencing were performed using standard methods to confirm cosegregation of candidate variants.

## Results

### Clinical findings

#### Family 1

A single affected female (IV-4) was the fourth child born to a consanguineous Pakistani couple (Fig. 1a), reported to have a severe seizure phenotype, poor vision, and profound psychomotor retardation (Table 1). Parents reported an uneventful antenatal, birth and neonatal history, with normal early development. An ocular phenotype of strabismus with nystagmus were the first symptoms described by the parents around 5 months of age. Onset of generalised tonic-clonic (GTC) seizures with excessive startle reflex were observed at 7 months of age with increasing frequency and severity over time, although control was improved after the introduction of phenobarbital. At 1 year of age loss of visual fixation was the earliest sign of regression. Over time further loss of vision, hearing, speech and motor skills progressed with loss of independent sitting and head control and no response to any stimuli by 18 months, associated with increased GTC seizure frequency. An older male sibling (IV-2) was reported by the parents to have died at the age of 18 months with a similar neurodegenerative phenotype.

**Table 1:** Clinical features of families 1, 2 & 3

FAMILY	1		2					3	
Individual	IV-2	IV-4	IV-3	IV-4	IV-5	IV-6	IV-7	IV-1	IV-2
Genotype	*HEXB* HOM c.445 + 1G > T	N/K	*MBOAT7* HOM c.758_778del	*MBOAT7* HOM c.758_778del	N/K	*MBOAT7* HOM c.758_778del	*MBOAT7* HOM c.758_778del	*MBOAT7* HOM c.758_778del	*MBOAT7* HOM c.758_778del
Sex	F	M	M	M	F	F	M	M	F
Age at assessment (years)	20 m	Not assessed (deceased at 18 m)	20	18	16	19	9	8y 1 m	12y
Gestation weeks	FT	N/K	FT	FT	N/K	N/K	FT	FT	FT
Birth weight kg (SD)	3.0 (−0.9)	N/K	3.0 (−1.16)	2.9 (− 1.38)	N/K	N/K	2.8	1.5 (−4.6)	1.8 (−3.86)
Height cm (SD)	89 (+ 2.27)	N/K	172.5 (−0.69)	168.8 (−1.2)	N/K	N/K	106.7 (−4.58)	106.7 (−3.93)	127 (−3.2)
Weight kg (SD)	8.5 (−2.67)	N/K	55 (−1.95)	68 (+ 0.09)	N/K	N/K	30 (+ 0.34)	N/K	N/K
Head circumference cm (SD)	51 (+ 2.2)	N/K	50 (−2.8)	55.5 (−1.03)	53.6 (− 1.25)	55.5 (−0.01)	54 (− 1.9)	49 (−3.11)	52 (− 1.81)
Development									
Intellectual disability	+	+	Mod-Severe	Severe	Mod-Severe	Mod-Severe	Mod-Severe	Mod-Severe	Mod-Severe
Speech delay/impairment	Non-verbal	+	Non-verbal	Non-verbal	+	+	Non-verbal	Non-verbal	Non-verbal
Developmental delay	+	+	+	+	+	+	+	+	+
Age walking	N/A	N/A	3.5y	4y	N/A	N/A	3.5y	>4y	4y
Neurological features									
Macrocephaly	+	N/K	–	–	–	–	–	–	–
Microcephaly	–	N/K	+	–	–	–	–	+	+
Seizures	Onset 7 m GTCS Regression	+	Onset 1.5y Focal/ multifocal infantile	Onset 2.5y Focal/ multifocal infantile	Febrile seizures in infancy	Febrile seizures in infancy	Febrile seizures in infancy	Onset in infancy GTCS	Onset in infancy GTCS
Hypotonia	+	N/K	+	+	+	+	N/K	+	+
Behavioural problems	N/A	N/A	–	Aggressive episodes	–	–	–	Aggressive episodes Hyperactivity	Aggressive episodes
Other features	Strabismus Nystagmus Visual loss Hearing loss Hepatosplenomegaly	Deceased aged 1.5 years			Unable to walk	Unable to walk			Reduced physical activity level

*F* female, *M* male, *HOM* homozygous, *FT* full-term, *N/A* Not applicable, *N/K* not known, *m* months, *y* years, +; feature present, −; feature absent, *GTCS* generalised tonic-clonic seizure

#### Family 2

Family 2 comprises five affected individuals (IV-3, IV-4, IV-5, IV-6 and IV-7) with global developmental delay, moderate to severe ID, hypotonia and behavioural problems, including aggression and hyperactivity (Table 1), in a large consanguineous (parents are first-cousins) Pakistani family (Fig. 1d) from Mardan city, a remote region of Khyber Pakhtunkhwa province of Pakistan. Two brothers (IV-3 and IV-4) had infantile focal and multifocal epilepsy with seizure onset at 1.5 and 2.5 years respectively, which has been responsive to antiepileptic medication. Furthermore, three affected individuals (IV-5, IV-6 and IV-7) developed febrile seizures in infancy. All subjects have a below average head size (Table 1), and one individual has microcephaly (IV-3); MRI brain imaging of this individual revealed mild diffuse cerebral atrophy with no sulcal prominence or ventricular enlargement.

#### Family 3

This family comprises two affected siblings (IV-1 and IV-2) with microcephaly, GTC seizures from infancy, moderate to severe ID, global developmental delay, including absent speech, poor memory, and behavioural problems including aggressive episodes and hyperactivity (Table 1), who were born to a consanguineous Pakistani couple (Fig. 1e) from Swat city of the Khyber Pakhtunkhwa province of Pakistan. An EEG performed on individual IV-1 at the age of 3 years showed a mixed background rhythm of beta and theta waves, with abnormal bursts of sharp waves and generalized slow waves on arousal from sedation. MRI brain imaging of this individual (IV-1) shows regions of cortical atrophy, coronal T2 image shows cortical thinning and loss of underlying white matter leading to enlarged fissures in the vermis and cerebellar hemispheres.

### Genetic findings

WES was performed using DNA from a single affected individual (IV-2) from family 1, after filtering variants for quality, zygosity, population frequency and predicted outcome, a single homozygous splice site variant (chr5:g.74689474G > T; c.445 + 1G > T [rs761197472]) was identified in *HEXB* (NM_000521, a gene previously associated with an autosomal recessive neurodegenerative disorder, for filtering steps in variant prioritization). Dideoxy sequencing confirmed cosegregation of this variant within Family 1 (Fig. 1a, b). This *HEXB* splice variant c.445 + 1G > T is predicted to affect the canonical splicing of exon 2 by abolishing the normal 5′ donor splice site, which is predicted to likely result in skipping of exon 2, and possibly promotes the use of a cryptic splice site upstream of the intronic 5′ donor sequence (Fig. 1c). This rare variant is present as heterozygous in a single South Asian individual in gnomAD (allele frequency 0.00003266), with no homozygous individuals. A splice variant at the same position (c.445 + 1G > A) has previously been published as a cause of disease in several patients from Argentinian families with SD [13–15] and is reported as pathogenic in ClinVar and the HGMD database. A further splice variant at this position (c.445 + 1G > C) is also listed in ClinVar and dbSNP with conflicting interpretations of pathogenicity.

Genome-wide SNP-array genotyping of DNA from affected individuals IV-3, IV-4 and IV-7 identified a single 1.7 Mb region of shared homozygosity between the affected individuals from rs465169 to rs2112834 (chr19: 54,023,718-55,785,242 [hg38], containing 82 protein coding genes. Of these genes only 9 had OMIM morbid phenotypes: *DNAAF3, GP6, KIR3DL1, MBOAT7, NLRP7, PRPF31, TNNI3, TNNT1 and TSEN34* and only two had associated neurological phenotypes compatible with that of affected individuals within this family: *TSEN34* [16] and *MBOAT7* [7]. *MBOAT7* variant assessment was prioritized as more literature has been reported on the phenotype associated with this gene, including two recently published variants identified within Pakistani families [7] (Table 2). Dideoxy sequencing of *MBOAT7* (NM_024298.3) using primers that cover both previously identified variants within the Pakistani population (c.820_826del p.(Gly274Profs*47) and c.758_778del; p.(Glu253_Ala259del)), revealed the 21 base pair in-frame deletion (Chr19:g.54180849_54180869del21; c.758_778del; p.(Glu253_Ala259del) [hg38] [rs750035706]) in exon 6 that cosegregated in family 2 (Fig. 1d, f-g). Due to an overlapping phenotype and common origin of families 2 and 3, we performed dideoxy sequencing for the c.758_778del *MBOAT7* variant in family 3, which confirmed cosegregation (Fig. 1e). The *MBOAT7* c.758_778del variant is reported in gnomAD in 5 heterozygous individuals, (four South Asian and one Finnish), with an allele frequency of 0.00002333 (0.0001571 in the South Asian population), and is listed in Clinvar and dbSNP as pathogenic. This variant results in loss of seven highly conserved amino acids (p.Glu253_Ala259del) [rs750035706] from the MBOAT7 protein thus producing a shorter protein, and is predicted to be pathogenic by in silico prediction tools. Neither of these identified variants in *HEXB* (c.445 + 1G > T) and *MBOAT7* (c.758_778del) were present in 65 Pakistani exomes of unaffected individuals.

**Table 2:** *MBOAT7* variants published to date associated with autosomal recessive neurodevelopmental disorder

Publications	Number of individuals	Number of families	Origin	Transcript	gDNA position [hg38]	cDNA position	Predicted Protein change	Exon	Type	gnomAD frequency (All)
Present paper	7	2	Pakistan	NM_024298.3	g.54180849_54180869del21	c.758_778del	p.Glu253_Ala259del	6	Inframe deletion	0.00002333
Johansen et al (2016)	5	2	Pakistan	NM_024298.3	g.54180849_54180869del21	c.758_778del	p.Glu253_Ala259del	6	Inframe deletion	0.00002333
Johansen et al (2016) [7]	4	1	Pakistan	NM_024298.3	g.54180801_54180807delGGCCGCC	c.820_826del	p.Gly274Profs*47	6	Frameshift	–
Johansen et al (2016) [7]	3	1	Egypt	NM_024298.3	g.54188278_54188297del20	c.126_145del	p.Leu43Hisfs*69	3	Frameshift	–
Johansen et al (2016) [7]	2	1	Jordan	NM_024298.3	g.54183591delC	c.423delG	p.Leu142Cysfs*8	5	Frameshift	–
Johansen et al (2016) [7]	2	1	Iraq	NM_024298.3	g.54180772C > G	c.854 + 1G > C	p.?	Intron	Splice	–
Hu et al (2018) [9]	3	1	Iran	NM_024298.3	g.54174394C > T	c.1069G > A	p.Gly357Ser	8	Missense	0.00001702
Santos-Cortez et al (2018) [8]	4	1	Pakistan	NM_024298.3	g.54187242delA	c.251delT	p.Leu84Argfs*25		Frameshift	–
Yalnizoglu et al (2019) [6]	3	1	Turkey	NM_024298.3	g.54174186G > A	c.1278G > A	p.Trp426*	8	Nonsense	–
Yalnizoglu et al (2019) [6]	4	2	Turkey	NM_024298.3	g.54162440_54174072del	c.?	p.?	8	Deletion	–
Yalnizoglu et al (2019) [6]	2	1	Turkey	NM_024298.3	g.54187234C > T	c.259C > T	p.Arg87Gln	4	Missense	0.00001108
Yalnizoglu et al (2019) [6]	2	2	Turkey	NM_024298.3	g.54180936_54180946del	c.680_690del	p.Leu227Profs*65	6	Frameshift	0.000004919
Yalnizoglu et al (2019) [6]	1	1	Turkey	NM_024298.3	g.54174337C > T	c.1126G > A	p.Glu376Lys	8	Missense	0.00003211
Jacher et al. (2019) [10]	1	1	Lebanon	NM_024298.3	g.54178943 T > C	c.855-2A > G	p.?	Intron	Splice	–

## Discussion

We investigated three extended consanguineous Pakistani families with individuals affected by undiagnosed childhood onset neurological disease, aiming to provide an accurate molecular diagnosis for these families. WES in a single affected individual in family 1 identified a homozygous splice variant (NM_000521; c.445 + 1G > T) in *HEXB* that segregated within the family and confirms a diagnosis of SD within this individual and her deceased sibling. The clinical features reported in this patient, including hypotonia, generalized tonic-clonic or myoclonic seizures with regression, blindness, psychomotor retardation, ID, macrocephaly, hepatosplenomegaly, and death in infancy are consistent with previous reports of SD [17, 18]. Many variants have been reported in *HEXB* associated with SD, including 116 pathogenic (DM) variants listed within the Human Gene Mutation Database (HGMDpro, http://www.hgmd.cf.ac.uk/ac/index.php). Disruption of the hexosaminidase beta subunit results in a deficiency of the enzymes hexosaminidases A and B, and results in the accumulation of GM2 ganglioside within neurons that results in progressive destruction of the CNS. This is the first description of the c.445 + 1G > T variant that we are aware of within the Pakistani population, although another splice variant at this position (c.445 + 1G > A) has previously been reported in Argentinian families with SD [13, 14].

Genome-wide SNP mapping was carried out using DNA from all three affected individuals from family 2 and identified a 1.7 Mb region of shared homozygosity (chr19: 54,023,718-55,785,242 [hg38]). This region includes the *MBOAT7* gene, recently reported by Johansen et al. (2016) [7] to be associated with a neurodevelopmental phenotype characterised by developmental delay/ID seizures, hypotonia, autistic features and below average head size. This group reported two different homozygous variants in *MBOAT7* within three consanguineous Pakistani families; a 7 bp frameshift deletion (c.820_826del [p.Gly274Profs*47]) in exon 6 in four affected individuals from a single family, and an in-frame deletion (c.758_778del [p.Glu253_Ala259del]) also in exon 6 in five affected individuals from two unrelated families. We identified the same 21 base pair in-frame deletion (c.758_778del; p.Glu253_Ala259del) in families 2 and 3, which cosegregated as appropriate for an autosomal recessive condition. Our findings in these families are consistent with the clinical features described previously (Tables 1 and 3), confirming a diagnosis of an *MBOAT7*-associated disorder in the patients presented here.

**Table 3:** Clinical features of all published cases associated with biallelic *MBOAT7* variants

Publications	Current study	Johansen et al [7]	Hu et al [9]	Santos-Cortez et al [8]	Yalnizoglu et al [6]	Jacher et al [10]	Total (*n* = 39)
Sex	M = 4, F = 3	M = 7, F = 9	M = 3, F = 0	M = 2, F = 2	M = 5, F = 7	M = 0, F = 1	M = 21, F = 22
Consanguinity	7/7 (100%)	16/16 (100%)	3/3 (100%)	4/4 (100%)	12/12 (100%)	0/1 (0%)	42/43 (98%)
Development							
Developmental delay	7/7 (100%)	16/16 (100%)	0/3 (0%)	4/4 (100%)	12/12 (100%)	1/1 (100%)	40/43 (93%)
Speech delay	7/7 (100%)	16/16 (100%)	0/3 (0%)	4/4 (100%)	12/12 (100%)	1/1 (100%)	40/43 (93%)
Non-verbal	5/7 (71%)	9/16 (56%)	0/3 (0%)	N/K	N/K	0/1 (0%)	14/27 (52%)
Single words	2/7 (29%)	7/16 (44%)	N/K	N/K	N/K	1/1 (100%)	10/24 (42%)
Two word sentences	0/7 (0%)	2/16 (13%)	N/K	N/K	3/12 (25%)	1/1 (100%)	6/36 (17%)
Motor delay	7/7 (100%)	16/16 (100%)	0/3 (0%)	N/K	12/12 (100%)	1/1 (100%)	36/39 (92%)
Never walked	0/7 (0%)	3/16 (19%)	0/3 (0%)	N/K	0/12 (0%)	0/1 (0%)	3/39 (8%)
Neurological features							
Intellectual disability	7/7 (100%)	16/16 (100%)	3/3 (100%)	4/4 (100%)	12/12 (100%)	1/1 (100%)	43/43 (100%)
Lower than average OFC	7/7 (100%)	16/16 (100%)	0/3 (0%)	4/4 (100%)	N/K	0/1 (0%)	27/31 (87%)
Microcephaly	2/7 (29%)	6/16 (38%)	0/3 (0%)	2/4 (50%)	N/K	0/1 (0%)	10/31 (32%)
Macrocephaly	0/7 (0%)	0/16 (0%)	0/3 (0%)	0/4 (0%)	0/7 (0%)	1/1 (100%)	1/43 (2%)
Seizures	6/7 (86%)	10/16 (63%)	3/3 (100%)	N/K	11/12 (92%)	1/1 (100%)	31/39 (79%)
Generalised tonic clonic	0/7 (0%)	1/16 (6%)	N/K	N/K	2/12 (17%)	0/1 (0%)	3/36 (8%)
Myoclonic/infantile spasm	0/7 (0%)	5/16 (31%)	N/K	N/K	3/12 (25%)	0/1 (0%)	8/36 (22%)
Focal	2/7 (29%)	2/16 (13%)	N/K	N/K	1/12 (8%)	1/1 (100%)	6/36 (17%)
Febrile seizures	2/7 (29%)	2/16 (13%)	N/K	N/K	1/12 (8%)	0/1 (0%)	5/36 (14%)
Hypotonia	6/7 (86%)	15/16 (94%)	N/K	N/K	12/12 (100%)	1/1 (100%)	34/36 (94%)
Hypertonia	0/7 (0%)	16/16 (100%)	N/K	N/K	0/12 (0%)	0/1 (0%)	16/36 (44%)
Behavioural problems/ASD	3/7 (43%)	7/16 (44%)	3/3 (100%)	N/K	4/12 (33%)	1/1 (100%)	18/39 (46%)
Poor coordination/ataxic gait	0/7 (0%)	0/16 (0%)	N/K	N/K	11/12 (92%)	N/K	11/35 (31%)
Neuroimaging	1/7 (14%)	6/16 (38%)	1/3 (33%)	N/K	12/12 (100%)	1/1 (100%)	21/39 (54%)
Polymicrogyria	0/1 (0%)	2/6 (13%)	0/3 (0%)	N/K	0/12 (0%)	0/1 (0%)	2/23 (9%)
Cortical atrophy	1/1 (100%)	2/6 (13%)	0/3 (0%)	N/K	8/12 (67%)	0/1 (0%)	11/23 (45%)
Cerebellar dysgenesis	0/1 (0%)	0/6 (0%)	0/3 (0%)	N/K	8/12 (67%)	0/1 (0%)	8/23 (35%)
Leukoencepahlopathy	0/1 (0%)	0/6 (0%)	1/1 (100%)	N/K	0/12 (0%)	1/1 (100%)	2/21 (10%)
Other features							
Strabismus	N/K	N/K	1/3 (33%)	N/K	5/12 (42%)	1/1 (100%)	7/16 (44%)
Retinal/macular degeneration	0/12 (0%)	N/K	2/3 (67%)	N/K	N/K	0/1 (0%)	2/16 (13%)
Optic atrophy	0/12 (0%)	N/K	3/3 (100%)	N/K	N/K	0/1 (0%)	3/16 (19%)
Hyperphagia/obesity	0/12 (0%)	N/K	3/3 (100%)	N/K	N/K	1/1 (100%)	4/16 (25%)
Short stature	3/7 (43%)	N/K	0/3 (0%)	N/K	N/K	0/1 (0%)	3/11 (27%)

Comparison of clinical features of all published cases of neurological disorder associated with biallelic *MBOAT7* variants, showing number and percentage of individuals (in brackets) with each feature. *OFC* occipitofrontal circumference, *ASD* autistic spectrum disorder, *N/K* not known

This study adds seven affected individuals from two Pakistani families to the literature, with a total of 43 individuals now described with biallelic pathogenic *MBOAT7* variants and similar overlapping phenotypes (Table 3). A total of 13 *MBOAT7* variants associated with autosomal recessive neurodevelopmental disorder have been described to date (Table 2); nine of which are truncating and loss-of-function variants and not tolerated in gene constraint predictions with a pLI score of 0.113 in gnomAD. A total of 12 individuals from four consanguineous Pakistani families with a similar neurodevelopmental phenotype have now been reported as homozygous for the *MBOAT7* c.758_778del variant (Table 2). The further two families reported here contribute to knowledge of the phenotypical spectrum of neurological disorder associated with disruption of MBOAT7, characterised by the universal feature of moderate to severe ID, usually associated with significant global developmental delay, profound speech impairment (52% are non-verbal), motor delay (8% never walk) and lower than average OFC (32% have microcephaly) (Table 3). Other frequent features are seizures in 79%, including GTC, myoclonic, infantile spasm, focal and multifocal seizures, hypotonia is reported in 94% (often described as truncal hypotonia in infancy), hypertonia is also reported in 44%, autistic features and behavioral problems include aggressive episodes, hyperactivity, stereotypies (rocking and hand flapping) in 46%. Jacher et al. [10] reported macrocephaly with overgrowth in a single patient, although macrocephaly has not been observed in any other previously reported cases. While we cannot exclude additional genetic or environmental causes our data suggest that short stature (below − 3 SD identified in 3/5 individuals) may also be a feature of *MBOAT7*-related disorder. MRI imaging identified several common findings of cortical atrophy in 45%, cerebellar dysgenesis in 35%, leukocencepahlopathy in 10% and polymicrogyria in 9% of individuals who underwent imaging and as previously discussed by *Yalnizoglu* et al [6], these findings are common to other complex lipid biosynthesis and remodeling disorders.

The *MBOAT7* gene encodes LPIAT1, an enzyme present in endomembranes that contributes to the regulation of free arachidonic acid (AA) in the cell through the remodeling of phospholipids via the Land’s cycle [19, 20]. Lee et al. [18] discovered that LPIAT1 is required for cortical lamination in *Mboat7*^*−/−*^ mice and brain histology of these mice showed a smaller cerebral cortex, with increased apoptotic cells and increased gyral structures. These findings are comparable with the phenotype observed in humans of cortical atrophy, reduced head size and polymicrogyria. Interestingly, *Mboat7*^*−/−*^ mice show significantly smaller stature than their wildtype littermates [21], and is a feature that we have identified in our patient cohort with 3/5 individuals with height < − 3 SD below the mean, confirming this as a novel feature associated with *MBOAT7*-related neurodevelopmental disorder.

## Conclusions

Interestingly both protein products of *MBOAT7* and *HEXB* genes are involved in metabolic disorders of lipid biosynthesis and remodeling within the brain, and this group of disorders are an important and often overlooked consideration in the differential diagnosis of neurodevelopmental disorders [22]. Investigation and identification of the genetic basis of neurodevelopmental disorders identified within the three Pakistani families reported here provide us with a better understanding of the spectrum of neurological disease and responsible gene variants present within this population to aid diagnosis in other families who may be affected by these conditions. Accurate molecular disease diagnosis allows a specific diagnosis to be provided to families and their clinicians to provide targeted management strategies, appropriate genetic counselling, improved carrier detection and the possibility of prenatal testing where available. Our findings highlight the *MBOAT7* c.758_778del variant as a cause of developmental delay/ID in the Pakistani population, and broaden knowledge of the phenotypical outcomes associated with *MBOAT7* gene variants.

## Data Availability

The datasets used and/or analysed during the current study are available from the corresponding author on reasonable request.

## Abbreviations

AA: Arachidonic acid
ExAC: Exome Aggregation Consortium
GM2: Gangliosidases
GnomAD: Genome Aggregation Data base
GTCS: Generalized tonic clonic seizure
*HEXA*: Hexosaminadase A
*HEXB*: Hexosaminadase B
HGMD: Human gene mutation data base
HOM: Homozygous
ID: Intellectual disability
LPIAT1: Lysophosphatidyl inositol aceyl transferase 1
*MBOAT7*: Membrane bound O-aceyltransferase family member 7
MRI: Magnetic Resonance Imaging
SD: Sandhoff disease
SNP: Single Nucleotide Polymorphism
WES: Whole Exome Sequencing
